# Risk Factors for Corneal Endothelial Decompensation after Penetrating Keratoplasty: A Population-Based Cohort Study

**DOI:** 10.3390/jcm13030718

**Published:** 2024-01-26

**Authors:** Hung-Chi Chen, Chia-Yi Lee, Yu-Ling Chang, Jing-Yang Huang, Shun-Fa Yang, Chao-Kai Chang

**Affiliations:** 1 Department of Ophthalmology, Chang Gung Memorial Hospital, Linkou, Taoyuan 333, Taiwan; mr3756@cgmh.org.tw; 2 Department of Medicine, Chang Gung University College of Medicine, Taoyuan 333, Taiwan; 3 Center for Tissue Engineering, Chang Gung Memorial Hospital, Linkou, Taoyuan 333, Taiwan; 4 Institute of Medicine, Chung Shan Medical University, Taichung 402, Taiwan; 5 Nobel Eye Institute, Taipei 115, Taiwan; 6 Department of Ophthalmology, Jen-Ai Hospital Dali Branch, Taichung 412, Taiwan; 7 Department of Medical Education, Cathay General Hospital, Taipei 106, Taiwan; 8 Department of Medical Research, Chung Shan Medical University Hospital, Taichung 402, Taiwan; 9 Department of Optometry, Da-Yeh University, Chunghua 515, Taiwan

**Keywords:** endothelial decompensation, penetrating keratoplasty, epidemiology, type 2 diabetes mellitus, cataract surgery

## Abstract

(1) Background: Endothelial decompensation is a common complication after penetrating keratopathy (PK), while the risk factors for endothelial decompensation after PK have not been fully elucidated. Consequently, we aim to investigate the possible risk factors for endothelial decompensation after PK. (2) Methods: This retrospective study was conducted using the National Health Insurance Research Database (NHIRD) of Taiwan. The main outcome was the development of endothelial decompensation after PK surgery. The effects of potential risk factors were compared between the patients with endothelial decompensation and the patients without endothelial decompensation via Cox proportional hazard regression, which produced the adjusted hazard ratio (aHR) and a 95% confidence interval (CI). (3) Results: Overall, 54 patients developed endothelial decompensation after PK surgery, with a ratio of 16.12 percent. The pre-existing type 2 diabetes mellitus (T2DM) (aHR: 1.924, 95% CI: 1.257–2.533, *p* = 0.0095) and history of cataract surgery (aHR: 1.687, 95% CI: 1.328–2.440, *p* = 0.0026) were correlated with the development of endothelial decompensation. In the subgroup analysis, the correlation between a history of cataract surgery and post-PK endothelial decompensation was more prominent in patients older than 60 years compared to their younger counterparts (*p* = 0.0038). (4) Conclusions: Pre-existing T2DM and a history of cataract surgery are associated with a higher incidence of post-PK endothelial decompensation.

## 1. Introduction

Penetrating keratoplasty (PK) is a surgery that can be used to manage corneal opacities or other conditions like ectasia affecting a significant portion of the stroma alone, or also involving endothelium [1]. PK has been largely replaced by endothelial transplantation (either Descemet’s stripping automated endothelial keratoplasty or Descemet’s membrane endothelial keratoplasty) for isolated endotheliopathies, but it is still performed in patients with aphakic or pseudophakic bullous keratopathy, or Fuchs endothelial corneal dystrophy, when chronic edema has led to significant stromal fibrosis, or when the surgeons do not have enough experience with posterior lamellar procedures which are more technically exigent [2,3]. In addition, PK is also performed as an urgent therapeutic option in severe infectious keratitis [4]. Although the uncorrected visual acuity can be improved to above 20/40 in about 50 to 60 percent of eyes undergoing PK, a residual refractive error, frequently with high astigmatism, is still a concern [5,6,7]. Furthermore, postoperative complications after PK surgery can still develop, such as persistent corneal epithelial defects, stitch ruptures, infectious keratitis, and post-PK glaucoma [8,9].

For decades, it has been documented that PK is associated with a substantial loss of corneal endothelial cells, likely due to unavoidable surgical trauma. Consequently, corneal donor cell density diminishes between 30% and 50% within the first year of follow-up, which has been observed even in recent studies [10,11,12]. Five years post-transplantation, a further reduction in ECD, ranging between 69% and 75%, has been observed [13]. In addition, it has also been identified that certain corneas experience a chronic and significant loss of endothelial cells, leading to the eventual development of late irreversible edema, which is also known as secondary failure. Endothelial decompensation is estimated to develop in between 5 and 9 percent of patients after PK surgery [14,15]. In these cases, the corneal endothelium demonstrates a nearly complete inhibition of in vivo mitosis, and there are no approved medical treatments yet for triggering endothelial cell regeneration. Although some promising options are under research, such as Rho kinase inhibitors, the only available recourse is a regraft—either a second PK or an endothelial transplantation [16,17,18,19,20]. There is an increased risk of immune rejection after a second PK, and it can present even following lamellar endothelial grafts in these cases [21].

From studies performed more than 25 years ago, endothelial decompensation after PK has been associated with factors, such as immune rejections, low endothelial cell density at 2 months post-surgery, aphakia or pseudophakia, and older recipient and donor age, among others [7,22,23]. A recent study also showed that old age and lower endothelial cell density (ECD) were risk factors for endothelial decompensation after PK surgery [24]. Additionally, graft size and a history of type 1 diabetes mellitus, as well as type 2 diabetes mellitus (T2DM), in donors correlated to the development of post-PK endothelial decompensation [25]. Still, the possible systemic and ocular disease factors for post-PK endothelial decompensation have seldom been evaluated. Since some systemic diseases, including T2DM and hyperlipidemia, could damage the cornea [26,27], there may be some systemic or ocular risk factors for post-PK endothelial decompensation that need validation.

Accordingly, the purpose of this study is to evaluate the potential systemic and ocular risk factors for post-PK endothelial decompensation. The data from the Taiwan National Health Insurance Research Database (NHIRD) were applied for statistical analysis in this study.

## 2. Materials and Methods

### 2.1. Data Source

The Taiwan NHIRD preserves the claimed data derived from the Taiwan Health Insurance System with the medical records of more than 23 million Taiwanese people. The NHIRD data were sourced from the period of 1 January 2000 to 31 December 2016. The data in the NHIRD involve the International Classification of Diseases—Ninth Revision (ICD-9) diagnostic code, International Classification of Diseases—Tenth Revision (ICD-10) diagnostic code, sex, age, inhabitant location, education level, urbanization, image exam codes, laboratory exam codes, medical department codes, procedure codes, surgery codes, and the international ATC codes for medications. In this study, the longitudinal health insurance database (LHID) 2000 version was adopted, which is a sub-database of the NHIRD that stores the data of about two million individuals that were randomly extracted by the built-in software of the NHIRD program in the year 2000. All the data in the NHIRD can be obtained from the LHID 2000, and the interval used by the LHID 2000 is the same interval as the Taiwan NHIRD, from 1 January 2000 to 31 December 2016. In our study, one author (J.-Y.H.) went to the location provided by the National Health Insurance Bureau to perform the data search and analysis. All the authors discussed the data and results together and a re-search/analysis was planned if our group concluded that the data or analyses were problematic, and our request was approved by the National Health Insurance Bureau.

### 2.2. Patient Selection

**Figure 1:**
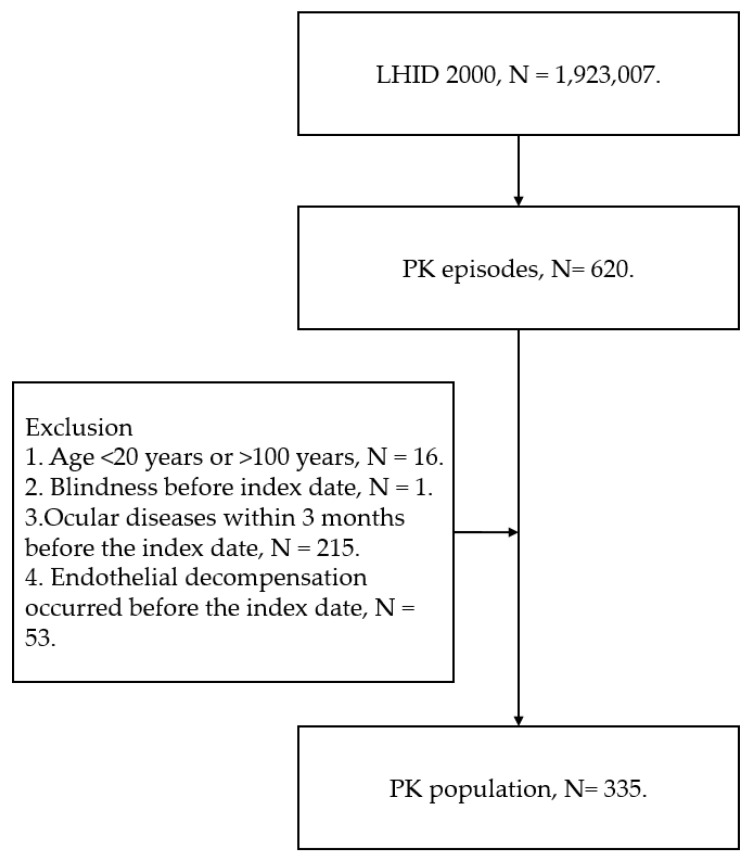
The flowchart of patient selection. LHID: longitudinal health insurance database, N: number, PK: penetrating keratoplasty.

The study employed a retrospective design involving all patients who underwent PK and complied with the inclusion criteria. Subsequently, the occurrence of postoperative endothelial decompensation was assessed. Adjusted hazard ratios were calculated for covariates, with one chosen as a reference to explore potential risk factors. Inclusion criteria required patients to meet the following conditions: (1) having undergone PK, as indicated by relevant surgery codes; (2) having received both topical antibiotics and steroids post-PK surgery, as identified by ATC codes; and (3) having the PK surgery performed by a qualified ophthalmologist with a degree, excluding procedures conducted by trainees. These criteria were established to ensure a homogeneous cohort with standardized surgical and postoperative care practices, enhancing the internal validity and reliability of the study results. The index date, which signifies the commencement of the observation period for each participant in the study and corresponds to the moment when the exposure or intervention of interest occurred, was set as the same date as the PK surgery. To increase the homogeneity of the PK population as much as possible, we utilized these exclusion criteria: (1) patients younger than 20 years or older than 100 years, (2) blindness recorded before index date, (3) ocular tumor recorded before index date, (4) infectious endophthalmitis recorded within 3 months before the index date, (5) dry eye disease recorded within 3 months before the index date, (6) epithelial defect recorded within three months before the index date, and (7) previous endothelial decompensation recorded before the index date. Because our aim was to evaluate primary endothelial decompensation after PK, patients with previous endothelial decompensation were beyond our consideration. Additionally, the purpose of excluding corneal diseases, ocular disorders, and blindness was to standardize the general ocular condition of our study population. After the process was complete, a total of 335 PK patients were included in this study and the subsequent analyses. The flowchart of patient selection is demonstrated in Figure 1.

### 2.3. Main Outcome

The main outcome in this study was set as endothelial decompensation that met the following conditions: (1) the presence of endothelial decompensation-related ICD-9 or ICD-10 diagnostic codes after the index date; (2) the arrangement of slit-lamp biomicroscope exams and specular microscope exams, as identified via the procedure codes; (3) the application of oral or topical corticosteroids after the endothelial decompensation diagnosis, as identified via the ATC codes; and (4) endothelial decompensation diagnosis and related medications made in an ophthalmic department. We tracked the patients in the Taiwan NHIRD until either the death of the participant, endothelial decompensation occurred, the patient withdrew from the National Health Insurance system, or the deadline of the NHIRD/LHID 2000 was reached (31 December 2016).

### 2.4. Risk Factors

To adjust the potential impact of demographic data, systemic co-morbidities, and ophthalmic disorders on endothelial decompensation development, we included these covariates in our analysis model: age, sex, urbanization level, hypertension, T2DM, ischemic heart diseases, hyperlipidemia, cerebrovascular disease, uveitis, history of retinal detachment with surgery, history of cataract surgery, and history of vitreous hemorrhage with surgery. The criteria for these confounding factors were determined according to the related demographic, ICD-9/ICD-10, and surgery codes in the LHID 2000. To ensure the intervals of systemic and ophthalmic diseases adequately influenced the incidence of endothelial decompensation, we only accounted for the systemic and ophthalmic co-morbidities/managements that lasted for longer than two years before the index date as our confounding factors.

### 2.5. Statistical Analysis

The SAS version 9.4 (SAS Institute Inc., Cary, NC, USA) was administered for statistical analyses of this study. Descriptive analyses were used to present the distributions of PK surgeries, demographic features, systemic disorders, and ocular diseases. Then, Cox proportional hazard regression was applied to produce the adjusted hazard ratios (aHRs) and associated 95% confidence intervals (CIs) of the demographic data, systemic morbidities, and ophthalmic disorders for comparisons between individuals with endothelial decompensation and individuals without endothelial decompensation. Of note, the influences of demographic data, systemic morbidities, and ophthalmic disorders were adjusted in the Cox proportional hazard regression. For the subgroup analyses, the participants were categorized via age (older than 60 years or not) and sex (male or female), and Cox proportional hazard regression was utilized again to investigate the influence of T2DM and a history of cataract surgery on the development of endothelial decompensation in each age and sex subgroup. Additionally, the interaction test was adopted to compare the correlations between T2DM or a history of cataract surgery and endothelial decompensation in different subgroups. Statistical significance was regarded as *p* < 0.05 in this study, and all the *p* values below 0.0001 were described as *p* < 0.0001 in this study to maintain a reasonable length.

## 3. Results

### 3.1. Basic Characteristics of Study Population

The clinical features of the study population who received PK surgery are shown in Table 1. A total of 186 (55.52%) male and 149 (44.48%) female patients were analyzed in this study. Most patients were aged from 70 to 79 years old (23.88%) and lived in urban areas (59.10%). The most prevalent systemic disease was hypertension, which developed in 112 (33.43%) individuals, and the most prevalent ophthalmic disease was cataracts; 24 (7.16%) patients received cataract surgery before PK surgery (Table 1).

### 3.2. Risk Factors for Endothelial Decompensation

A total of 54 patients developed endothelial decompensation after PK surgery, with a ratio of 16.12 percent (Table 2). The mean number of days from PK to endothelial decompensation was 127.17. After adjusting for the possible confounding factors, pre-existing T2DM (aHR: 1.924, 95% CI: 1.257–2.533, *p* = 0.0095) and history of cataract surgery (aHR: 1.687, 95% CI: 1.328–2.440, *p* = 0.0026) were significantly correlated with the development of endothelial decompensation (Table 3). The other demographic data, systemic diseases, and ophthalmic diseases did not illustrate significant associations with post-PK endothelial decompensation (all *p* > 0.05) (Table 3).

### 3.3. Subgroup Analysis Stratified by Age and Sex

In the subgroup analysis, the existence of T2DM showed a significant association with the development of post-PK endothelial decompensation in all the age and sex subgroups (all *p* < 0.05). A history of cataract surgery also showed a significant association with post-PK endothelial decompensation in both sex subgroups (both *p* < 0.05). In terms of the age-stratification analysis, a history of cataract surgery demonstrated a significant correlation with post-PK endothelial decompensation in patients older than 60 years (aHR: 2.017, 95% CI: 1.518–2.925, *p* = 0.0002) but not in the population younger than 60 years (aHR: 1.262, 95% CI: 0.996–1.684, *p* = 0.0578). In addition, the correlation between a history of cataract surgery and post-PK endothelial decompensation was more prominent in the patients aged older than 60 years than their younger counterparts according to the interaction test (*p* = 0.0038) (Table 4).

## 4. Discussion

In this study, post-PK endothelial decompensation was associated with pre-existing T2DM and having a history of cataract surgery. In addition, the effect of T2DM on the development of post-PK endothelial decompensation was significant in all the age and sex subgroups. On the other hand, the patients who received cataract surgery before the PK surgery showed a statistically significant higher chance of developing post-PK endothelial decompensation, both in males and females and in patients older than 60 years.

There are several mechanisms that contribute to corneal endothelial damage and endothelial decompensation [28,29]. Fuchs’ corneal endothelial dystrophy, a heterogenous genetic eye condition with environmental factors playing a role, can damage the corneal endothelium through mechanisms involving increased oxidative stress and an abnormal microenvironment in Descemet’s membrane due to aberrant extracellular matrix deposition contributing to progressive corneal endothelial cell loss and dysfunction, corneal transplantation may be needed for these patients [3,30,31]. Additionally, infections can impair the corneal endothelium; for example, cytomegalovirus infection can lead to corneal endotheliitis with reduced ECD [32]. The herpes simplex virus is another etiology of corneal endotheliitis which requires antiviral agents to prevent endothelial decompensation [33]. On the other hand, anterior uveitis can also insult the corneal endothelium and repeated corneal transplantation may be needed [34]. In addition to the aforementioned etiologies, it has been recognized for decades that phacoemulsification, currently the standard method for cataract surgery, can lead to reduced endothelial cell density (ECD) and even to endothelial decompensation. The extent of endothelial cell loss is variable but has been frequently reported to be higher than 5%. A study published in 2023, utilizing intracameral moxifloxacin and dexamethasone, found a mean ECD decrement of 6.2% [35]. Additionally, an analysis of selected studies published between 2015 and 2021 on postoperative corneal endothelial cell loss following phacoemulsification with intracameral antibiotics, which have been increasingly used in recent years, revealed that the reported means of endothelial cell loss ranged between 3.6% and 14.5% [35]. Moreover, corneal decompensation, as well as bullous keratopathy after cataract surgery, is a major indication for PK surgery [4]. The pathophysiology of endothelial injury in cataract surgery may be due to the effects of ultrasound power and the oxidative stress produced during phacoemulsification [36]. Additionally, several systemic diseases, including T2DM, dyslipidemia, and cardiovascular diseases, were shown to lead to prominent corneal damage in previous publications [26,27,37]. As already mentioned, PK surgery is linked to significant loss of corneal endothelium of donor cornea which ranged between 30 and 50% reduction two years after surgery, and up to 75% five years after PK. Furthermore, endothelial decompensation and graft failure accounted for up to 10 percent of patients who received PK surgery [15]. Risk factors related to post-KP endothelial cell loss have been previously identified [10,11,12,13,14,24,25], and the concept is also supported by the findings of the present study.

In this study, patients with pre-existing T2DM and a history of cataract surgery showed a higher incidence of post-PK endothelial decompensation compared to the individuals without these conditions. In a previous study, the risk factors for post-PK endothelial decompensation included a longer death-to-excision time of donor cornea, lower ECD, previous T2DM history of the donor, and old age [14,24,25]. However, whether the diseases of the recipient alter the risk of post-PK endothelial decompensation has not been fully investigated. To our knowledge, this may be a preliminary experience that demonstrates the possible correlation between pre-existing co-morbidities, including pre-existing T2DM and a history of cataract surgery, and post-PK endothelial decompensation. In addition, the effect of each parameter was adjusted in the Cox proportional hazard regression, and thus, pre-existing T2DM and a history of cataract surgery may be independent risk factors for the development of post-PK endothelial decompensation. T2DM can influence various ocular tissues including the cornea and the crystalline lens [38,39]. Regarding the corneal endothelium and T2DM, the findings have not been consistent for its impact on ECD in studies performed over the last 40 years, although most of the research has found a significant reduction in ECD in comparison to healthy age-matched controls in patients diagnosed with T2DM [40,41,42,43,44,45,46,47]. In some studies, reduced ECD, poor pump ability of the corneal endothelium, and a thicker cornea were found in patients with T2DM [40,45]. Nevertheless, other studies have proposed that T2DM does not affect corneal endothelial morphology in individuals with good glycemic status [42,43]. Accordingly, it is possible that T2DM correlates with a higher risk of endothelial decompensation. On the other hand, cataract surgery can lead to endothelial decompensation, bullous keratopathy, and possible corneal opacity [48]. Although the donor cornea does not undergo the possible damage that the recipient experiences, we speculate that the damage from previous cataract surgery in the recipient might cause a worse corneal condition, and the wound-healing process in such corneas after PK surgery may be worse than those without a history of cataract surgery.

Of the factors that were not associated with the development of post-PK endothelial decompensation, uveitis adversely affects the corneal endothelium [49]. We speculate that since PK surgery is not performed until the quiescence of uveitis and anti-inflammatory medication can be applied for the possible postoperative inflammation caused by PK surgery [9], the activation of previous uveitis after PK may be uncommon. Vitrectomy, which is used to manage retinal detachment and vitreous hemorrhage, can cause corneal endothelial loss [50], but a previous vitrectomy may have less of an effect on the corneal endothelium after PK surgery. Still, we did not exclude pre-existing glaucoma, while anti-glaucomatous medications like preoperative topical carbonic anhydrase inhibitors and filtrating surgery applied in pre-existing or subsequent glaucoma have been strongly and universally associated with corneal decompensation in the earlier literature [51,52,53,54]. Furthermore, both anterior segment trauma and anterior synechiae may damage the corneal endothelium [52,54]. There was no record of previous trabeculectomy or filtrating surgery in our study population, and thus the degree of glaucoma (if it existed) may not have been very severe. Additionally, topical therapy with carbonic anhydrase inhibitors is not the first-line therapy for glaucoma management in Taiwan. As a result, the ratio of topical anti-glaucomatous medication and filtrating surgery-related corneal decompensation may not be high. Still, we cannot access the data on the exact IOP value and the existence of anterior segment trauma in the Taiwan NHIRD, nor the data on the development of endothelial decompensation and post-PK filtrating surgery concurrently, due to the design of the Taiwan NHIRD. Accordingly, subsequent instances of glaucoma and anterior segment deformity could affect our results, and a further study is advocated to evaluate these issues.

In the subgroup analyses, pre-existing T2DM demonstrated a significant correlation with subsequent endothelial decompensation in all the age and sex subgroups. There is sparse research that shows this association. T2DM is a major cause of various ocular surface diseases, including superficial keratitis and dry eye disease [55]. Additionally, epithelial defects are commonly associated with T2DM [27]. According to the results of this study, T2DM may have a universal effect on post-PK endothelial decompensation regardless of age and sex. The interaction test revealed no difference in the correlation between T2DM and post-PK endothelial decompensation between the age and sex subgroups, which further supports our hypothesis. A history of cataract surgery showed similar and significant associations with post-PK endothelial decompensation in the sex subgroups, while the correlation between a history of cataract surgery and post-PK endothelial decompensation was not prominent in the patients aged younger than 60 years. A possible reason may be that patients younger than 60 years old usually have softer cataracts which require less phaco-power, thus generating less oxidative stress in endothelial cells, and old age itself is a significant risk factor for endothelial loss and decompensation [24,52]. Consequently, younger patients demonstrate better endothelial recovery from cataract surgery [30], and the risk of post-PK endothelial decompensation is relatively lower in such a population compared to the elderly.

Concerning the epidemiology of T2DM and cataracts, T2DM is a common disease with an estimated incidence of 415 million patients in 2015, which may rise to 642 million by the year 2040 [56]. The existence of T2DM is related to a huge economic burden, as the co-morbidities of T2DM contribute to considerable mortality and morbidity statistics [56]. On the other hand, cataracts are also a prevalent disease and are the most common cause of blindness throughout the world; it is estimated that cataracts impair the visual acuity of 95 million individuals [57]. Cataract surgery is the most common ophthalmic surgery performed in recent decades, and the cost of cataract surgery is tremendous [57]. Since both T2DM and cataracts that warrant surgery are major diseases, whether they are associated with the major postoperative complications of PK should be demonstrated.

There are some limitations in this study. Firstly, we used the mentioned database rather than clinical data for the analyses in this study, and thus several critical indexes, including the indication of PK surgery, the quality and other details of donor corneal tissue, the details of PK surgery, the postoperative visual acuity of PK surgery, the external eye images after PK surgery, the endothelial decompensation severity including the percentage of endothelial loss, the specular and immunological/histopathological results, the severity of co-morbidities, the degree of cataracts, and the details of cataract surgery, could not be obtained. Secondly, only 335 patients were enrolled in this study, which may cause bias, and the fact that we did not separate the causes of endothelial decompensation into primary donor graft failure and secondary donor graft failure could have compromised our statistical analysis. In addition, because the tracking system of the NHIRD can only trace one outcome, we cannot know whether another PK operation was performed to manage post-PK endothelial decompensation. Finally, nearly all the participants in this study were Taiwanese, and thus, the external validity of this study is reduced.

## 5. Conclusions

In conclusion, the presence of T2DM in the recipient and a history of cataract surgery are significant risk factors in post-PK endothelial decompensation. This correlation was observed after adjusting for various confounders. Furthermore, the impact of DM on post-PK endothelial decompensation was observed across all age groups. However, the influence of a history of cataract surgery was more pronounced in patients aged 60 years and older. Consequently, for individuals with a history of T2DM or prior cataract surgery scheduled for PK, it might be recommended to consider donor corneas with higher endothelial densities. Additionally, implementing very rigorous preventive intraoperative measures and a more intensive postoperative anti-inflammatory treatment regimen may be advisable. A further large-scale prospective study to survey the effects of T2DM and a history of cataract surgery on the severity of post-PK endothelial decompensation is essential.

## Figures and Tables

**Table 1:** The clinical features of the study population.

Features	Study Population (N = 335)
Sex	
Male	186
Female	149
Age	
20–39	47
40–49	38
50–59	65
60–69	77
70–79	80
≥80	30
Urbanization	
Urban	198
Sub-urban	98
Rural	39
Systemic morbidities	
Hypertension	112
T2DM	67
Chronic ischemic heart diseases	31
Hyperlipidemia	59
Cerebrovascular disease	28
Ophthalmic morbidities	
Retinal detachment	13
Vitreous hemorrhage	7
Uveitis	23
Cataract surgery	24
Vitrectomy	9

T2DM: type 2 diabetes mellitus, N: number.

**Table 2:** Incidence risk of endothelial decompensation in the study population.

Results	N
Follow-up person-months	19,718
Median of follow-up months (interquartile range)	66.24 (25.33 to 118.67)
Mean days from PK to endothelial decompensation	127.17
Endothelial decompensation (percentage)	54 (16.12)
No endothelial decompensation (percentage)	281 (83.88)

N: number.

**Table 3:** The adjusted hazard ratio for endothelial decompensation in each covariate.

Covariate	aHR	95% CI	*p* Value
Sex			
Male	Reference		
Female	1.036	0.688–1.558	0.8664
Age			
20–39	0.602	0.261–1.392	0.2355
40–49	Reference		
50–59	0.583	0.284–1.199	0.1422
60–69	1.007	0.493–2.057	0.9851
70–79	1.368	0.670–2.792	0.3895
≥80	1.522	0.614–3.777	0.3647
Urbanization			
Urban	Reference		
Sub-urban	1.136	0.736–1.754	0.5638
Rural	0.904	0.449–1.821	0.7779
Systemic morbidities			
Hypertension	0.900	0.585–1.385	0.6322
T2DM	1.924	1.257–2.533	0.0095 *
Ischemic heart diseases	1.196	0.643–2.225	0.5716
Hyperlipidemia	0.730	0.414–1.289	0.2785
Cerebrovascular disease	0.784	0.393–1.562	0.4887
Ophthalmic morbidities			
Retinal detachment	2.300	0.863–6.131	0.0959
Vitreous hemorrhage	1.768	0.805–3.254	0.1084
Uveitis	0.939	0.441–2.003	0.8714
Cataract surgery	1.687	1.328–2.440	0.0026 *
Vitrectomy	1.765	0.450–6.916	0.4151

aHR: adjusted hazard ratio, CI: confidence interval, T2DM: type 2 diabetes mellitus, N: number. * denotes a significant correlation of covariate with endothelial decompensation.

**Table 4:** The effect of diabetes mellitus and history of cataract surgery on post-penetrating-keratoplasty endothelial decompensation stratified by age and sex.

Subgroup	aHR	95% CI	*p* Value
Age < 60 years			
T2DM	1.645	1.103–2.428	0.0234 *
Cataract surgery	1.262	0.996–1.684	0.0578
Age > 60 years			
T2DM	2.334	1.359–2.914	0.0071 *
Cataract surgery	2.017	1.518–2.925	0.0002 *^,#^
Male			
T2DM	1.997	1.233–2.568	0.0098 *
Cataract surgery	1.554	1.184–2.375	0.0029 *
Female			
T2DM	1.856	1.284–2.465	0.0090 *
Cataract surgery	1.703	1.406–2.619	0.0017 *

aHR: adjusted hazard ratio, CI: confidence interval, T2DM: type 2 diabetes mellitus. * denotes significant correlation of covariate with endothelial decompensation. ^#^ denotes higher incidence of endothelial decompensation compared to the patients who were aged below 60 and received cataract surgery.

## Data Availability

The original data used in this study are not available due to the policy of the National Health Insurance Administration.
